# Differential IgG4-Producing Plasma Cell Infiltration in Non- and Post-Transplant Plasma Cell Hepatitis

**DOI:** 10.3389/ti.2022.10182

**Published:** 2022-03-18

**Authors:** Brian H. Horwich, Tom Z. Liang, Jennifer L. Dodge, Shefali Chopra, Jeffrey A. Kahn, Takeshi Saito

**Affiliations:** ^1^ Department of Medicine, Keck School of Medicine, University of Southern California, Los Angeles, CA, United States; ^2^ Department of Pathology, Keck School of Medicine, University of Southern California, Los Angeles, CA, United States; ^3^ Division of Gastrointestinal and Liver Diseases, Department of Medicine, Keck School of Medicine, University of Southern California, Los Angeles, CA, United States; ^4^ Department of Population and Public Health Sciences, Keck School of Medicine, University of Southern California, Los Angeles, CA, United States; ^5^ USC Transplant Institute, Keck School of Medicine, University of Southern California, Los Angeles, CA, United States; ^6^ Department of Molecular Microbiology and Immunology, Keck School of Medicine, University of Southern California, Los Angeles, CA, United States; ^7^ USC Research Center for Liver Diseases, Keck School of Medicine, University of Southern California, Los Angeles, CA, United States

**Keywords:** plasma cell hepatitis, alloimmunity, autoimmunity, IgG4, autoimmune hepatitis

## Abstract

Autoimmune hepatitis (AIH), post-transplant recurrent AIH (rAIH), and plasma cell-rich rejection (PCR) are clinical diagnoses with the shared histopathologic hallmark of plasma cell hepatitis (PCH). As these histologically and serologically indistinguishable diagnoses are differentiated by clinical context, it remains uncertain whether they represent distinct immunologic phenomena. Improved understanding of immunoglobulin subclass 4-producing plasma cells (IgG4-PC) has brought attention to IgG4 as an immunophenotypic biomarker. To date, degree and clinical significance of IgG4-PC infiltration in PCH remain elusive. This retrospective, single-center study assessed IgG4-PC infiltration in AIH, rAIH, and PCR via standardized immunohistochemistry analysis. Identified cases from 2005 to 2020 (*n* = 47) included AIH (treatment-naïve AIH (tnAIH): n = 15 and AIH-flare on treatment (fAIH); *n* = 10), rAIH (*n* = 8), and PCR (*n* = 14) were analyzed and correlated with clinical characteristics. IgG4-Positivity (# IgG4-PC/# pan-IgG-expressing cells) distribution was heterogenous and overlapping [tnAIH: 0.060 (IQR 0.040–0.079), fAIH: 0.000 (0.000–0.033), rAIH: 0.000 (0.000–0.035), PCR: 0.228 (0.039–0.558)]. IgG4-Positivity was inversely correlated with corticosteroid use (*p* < 0.001). IgG4-Positivity ≥0.500 was associated with rapid AST improvement (*p* = 0.03). The variable IgG4-Positivity of AIH, rAIH and PCR suggests diverse and overlapping immunopathologic mechanisms and that current diagnostic schemes inadequately capture PCH immunopathology. We propose incorporation of IgG4-Positivity to refine current PCH classification and treatment strategies.

## Graphical Abstract

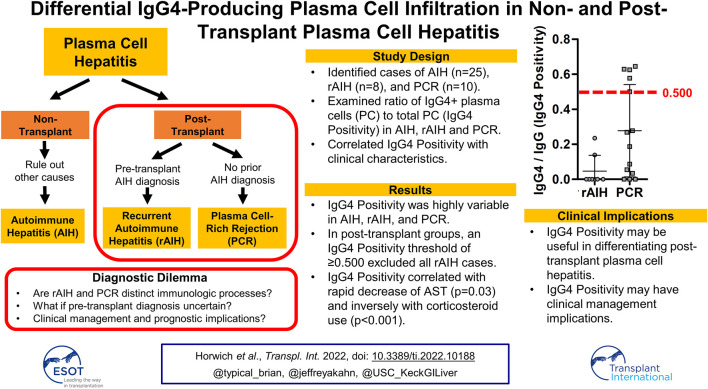

## Introduction

Plasma cell hepatitis (PCH) is a pathohistological finding characterized by lymphoplasmacytic portal and lobular inflammation with prominent plasma cells and often with the presence of interface hepatitis, perivenulitis and centrilobular necrosis (1–4). While the term “PCH” was originally used to describe autoimmune hepatitis (AIH), its use has extended to other plasma cell (PC)-rich necroinflammatory disorders including recurrent AIH (rAIH) and PC-rich rejection (PCR) in liver allograft (2, 5–7). Accordingly, the Banff Working Group defines that AIH represents PCH of the native liver, while rAIH and PCR are clinical entities of PCH occurring in the post-LT setting (8–12).

PCH often results in the development of hepatic fibrosis if not promptly and adequately treated with potent immunosuppressants (IS) such as corticosteroids (CS), antimetabolites, and calcineurin inhibitors (13–16). Moreover, IS often fail to sufficiently control hepatic necroinflammation, which eventually leads to liver failure (7, 14, 17, 18). Furthermore, the long-term administration of IS is associated with significant morbidity, including the development of opportunistic infections and neoplasms (19).

Since histopathological and serological assessment do not distinguish between AIH, rAIH, and PCR, their diagnosis is entirely reliant on clinical context, which is coupled with challenges, perhaps ambiguity (8, 12, 20–23). This is especially relevant in differentiating between rAIH and PCR. By convention, PCR refers to PCH in individuals who underwent transplantation for diseases other than AIH (8). Conversely, rAIH refers to PCH occurring in patients transplanted for AIH. Thus, based upon current convention, differentiating between rAIH and PCR may not be plausible in circumstances where pre-LT diagnosis was uncertain (e.g., acute liver failure of unknown etiology or cryptogenic cirrhosis). Furthermore, current PCH classification scheme excludes individuals transplanted for AIH from receiving a diagnosis of PCR. Thus, it remains elusive if rAIH and PCR represent distinctive or overlapping clinical entities. Similarly, there has not been evidence demonstrating that the onset of rAIH is mediated through the recurrence of immunopathology underlying AIH in the native liver, making the nomenclature of rAIH potentially deceiving. Ultimately, the fundamental issue regarding the current classification of PCH is the substantial degree of uncertainty as to whether each disease—as currently classified—represents a unique immunologic phenomenon**.**

PC, the terminally differentiated B cells, play a major role in the regulation of humoral immunity through the production of immunoglobulin (Ig). PC exhibit highly diverse immunomodulatory effects depending on the classes and subclasses of Ig production such as IgG, A, and M as well as IgG1, 2, 3, and 4, respectively (24). Hence, the Ig classes and subclasses expressed in the infiltrating PC would associate with, at least in part, the immunopathological presentations of PC-mediated disorders (24, 25). In particular, inflammatory disorders with a pronounced infiltration of IgG4-PC have been known to manifest marked tissue fibrosis and favorable response to IS (26). Therefore, chronic inflammatory disorders with IgG4-PC infiltration have emerged as a unique clinical entity, namely IgG4-related diseases (IgG4-RD) (27). The pancreas was the first organ in which IgG4-RD was recognized, namely autoimmune pancreatitis; thereafter this disease entity has been known to affect multiple organs, including the liver parenchyma (27, 28).

Prior studies have demonstrated the infiltration of IgG4-PC in the liver tissue of PCR and native-liver AIH, with a PCR subtype demonstrating significant enrichment (28, 29). Consequently, the Banff Working Group recommends use of IgG4 immunostaining in the evaluation of post-LT PCH (12). However, this recommendation lacks a specific threshold for IgG4-PC positivity and does not provide guidance with respect to its clinical relevance. One potential reason for this is that there has not been a comprehensive study that cross-sectionally compares the degree of IgG4-PC infiltration between PCH types with a standardized quantification method, significantly limiting its practical use. In particular, the degree of IgG4-PC infiltration in rAIH has not previously been studied. Thus, it remains elusive whether assessment of IgG4 immunostaining may be of diagnostic and therapeutic relevance in the post-LT setting—particularly in differentiating between rAIH and PCR. The primary aim of this study is to characterize PCH diseases by objectively determining the IgG4-PC positivity and evaluate for associations with clinical presentations and outcomes.

## Materials and Methods

### Study Subject Identification

All study procedures were approved by the University of Southern California Institutional Review Board (HS-19-00258). The study subjects were identified with the Department of Pathology Database by querying for reports containing “AIH,” “PCR”, or “PCH” from 2005 to 2020. The medical record and histopathological finding of all subjects identified through the database were confirmed to meet the diagnostic criteria of AIH (International Autoimmune Hepatitis Group) or PCH (Banff consensus) (12, 30, 31). Individuals with infectious and neoplastic etiologies as the cause of PC infiltration were excluded. All cases enrolled into this study were classified as the following: treatment-naïve AIH (tnAIH), AIH flare while on IS (fAIH), rAIH, and PCR. A diagnosis of tnAIH was defined as meeting probable or definite diagnostic criteria for AIH with no known IS use with activity against AIH prior to biopsy. A diagnosis of fAIH was defined as having a pre-existing diagnosis of AIH on IS with histopathology demonstrating features consistent with recurrence of active AIH disease. A diagnosis of rAIH was defined as allograft histopathology demonstrating features consistent with active AIH disease for which the pre-transplant diagnosis was AIH. A diagnosis of PCR was defined as clinical history and histopathology consistent with criteria outlined by the Banff Working Group (12). No specimen from executed prisoners were used.

### Clinical Data Collection

The medical records of identified subjects were reviewed to extract relevant demographic and clinical information.

### Specimen Processing and Histopathologic Review

The Formalin-Fixed Paraffin-Embedded (FFPE) liver needle core biopsy specimens of all enrolled cases (*n* = 47) were retrieved. The hematoxylin-eosin (H&E)-stained pathology slides were first reviewed for adequacy of the tissue as determined by 8 or greater portal tracts and at least 1.5 cm in length. All retrieved samples were deemed adequate by these criteria. For individuals with multiple biopsies with the same diagnosis, the initial biopsy specimen available was used. Cases were evaluated for the following histological characteristics: portal inflammation, interface lobular necro-inflammatory activity, perivenular inflammation and the presence of bridging necrosis. Both activity and fibrosis were assessed on the Metavir histological activity and fibrosis score. Any additional pathological findings were also recorded. The tissue specimens were serially sectioned and applied for immunohistochemical stains using anti-human pan-IgG (RWP49 clone: Leica Biosystems) at 1:1,000 dilution and anti-human IgG4 (MRQ-44 from Cell Marque) at 1:500 dilution. All staining was performed using Bond III Leica Autostainer system at the Human Pathology Core. The representative portal tract of each subject identified by an expert pathologist was used to determine the IgG4-Positivity, which was calculated by the number of IgG4 staining positive PC normalized by the number of pan-IgG staining positive PC in the corresponding portal tract of the serially sectioned slides. Counting was done manually on ×400 of both the IgG and IgG4 cells in that portal tract.

### Statistical Analysis

Demographic, laboratory, and histopathologic characteristics were summarized as medians with interquartile ranges (IQR) or frequencies with proportions for the overall cohort and stratified by PCH subtype. Characteristics were compared within the respective non-transplant (tnAIH versus fAIH) and transplant (rAIH versus PCR) groups using Wilcoxon-rank sum and Fisher’s exact tests, as appropriate.

Measures of IgG4 were characterized as the presence or absence of IgG4-PC and the proportion of IgG4-PC of all IgG-producing cells, which were compared by PCH subtype. The proportion of IgG4-PC was plotted by PCH subtype and separately categorized in tertiles to accommodate the skewed distribution. Laboratory parameters and therapeutic outcomes were evaluated for their association with increasing tertile of IgG4-Positivity using non-parametric trend tests (Stata nptrend) and markedly high IgG4-Positivity in the post-LT groups (≥0.500 vs. <0.500) using Kruskal-Wallis tests (Stata).

Differences were considered statistically significant at *p* < 0.05. Statistical analysis was conducted using SAS 9.4 (SAS Institute Inc., Cary, NC, United States) and Stata 14.0 (StataCorp, College Station, TX, United States).

## Results

### Demographics and Clinical Data

The demographics and clinical characteristics of the study subjects are summarized (Table 1). There was no subject crossover between groups. Median age was lowest for rAIH at 35 years compared to 50 or more years for the other PCH subtypes. All groups demonstrated a female predominance (tnAIH 73%, fAIH 60%, rAIH 87%, PCR 79%). Median BMI ranged from 24.3 to 29.5 (tnAIH 26.8, fAIH 24.3, rAIH 28.8, PCR 29.5), with 38% individuals having BMI>30 at time of biopsy. A family history of autoimmune disorders was more prevalent in the AIH groups (tnAIH 27%, fAIH 20%, rAIH 25%) when compared to PCR (0%). The most common etiologies of pre-transplant liver disease for the PCR group were non-alcoholic steatohepatitis (29%) and acute liver failure of indeterminate etiology (21%).

**TABLE 1:** Characteristics of individuals with plasma cell hepatitis.

Parameter	All subjects		Non-transplant		Transplant		
		tnAIH	fAIH	*p*-value	rAIH	PCR	*p*-value
	*N* = 47	*N* = 15	*N* = 10		*N* = 8	*N* = 14	
Age (years), median	51	52	50	0.70	35	52	0.22
Female, *n* (%)	35 (74)	11 (73)	6 (60)	0.67^a^	7 (87)	11 (79)	1.00^a^
BMI (kg/m^2^), median	29.1	26.8	24.3	0.74	28.8	29.5	0.86
Alcohol use, *n* (%)	9 (19)	8 (53)	0 (0)	**0.008** ^a^	0 (0)	1 (7)	1.00^a^
Drug use, *n* (%)	3 (6)	1 (7)	0 (0)	1.00^a^	1 (12)	1 (7)	1.00^a^
Family history of autoimmune diseases, *n* (%)	8 (17)	4 (27)	2 (20)	1.00^a^	2 (25)	0 (0)	0.12^a^
Liver disease prior to the liver transplantation, *n* (%)							
Acute liver failure					0 (0)	3 (21)	
Alcohol					0 (0)	0 (0)	
Autoimmune hepatitis					8 (100)	0 (0)	
Cryptogenic					0 (0)	2 (14)	
HBV					0 (0)	2 (14)	
HCV					0 (0)	1 (7)	
NASH/NAFLD					0 (0)	4 (29)	
Other					0 (0)	2 (14)	
Ethnicity, *n* (%)							
White	13 (28)	3 (20)	4 (40)		2 (25)	4 (29)	
Hispanic	3 (6)	2 (13)	0 (0)		0 (0)	1 (7)	
Asian	7 (15)	3 (20)	1 (10)	0.57^a^	0 (0)	3 (21)	0.37^a^
Black	6 (13)	2 (13)	0 (0)		3 (37)	1 (7)	
Other	18 (38)	5 (33)	5 (50)		3 (37)	5 (36)	
Time from Transplant (mo)							
Median		N/A	N/A		9.0	5.6	0.22
Range					2.6–24.3	0.2–25.6	
Immunosuppressants, *n* (%)							
CS	21 (45)	—	7 (70)		7 (87)	7 (50)	0.17^a^
CNI	22 (47)	—	2 (20)		7 (87)	13 (93)	1.00^a^
AZA	3 (7)	—	2 (20)		1 (12)	0 (0)	0.36^a^
MMF	13 (28)	—	0 (0)		3 (37)	10 (71)	0.19^a^
MTOR	1 (2)	—	0 (0)		1 (12)	0 (0)	0.36^a^
Laboratory Data^b^							
Platelet count (K/cumm), median (normal 141–401)	181	202	195	0.95	143	152	0.86
ALP (U/L), median (normal 34–106)	216	155	167	0.68	204	224	0.68
ALT (U/L), median (normal 14–54)	300	349	200	0.29	56	181	0.09
AST (U/L), median (normal 38–126)	306	366	147	0.19	76	155	0.19
TB (mg/dl), median (normal 0.2–1)	5.1	5.3	1.4	**0.03**	1.3	1.1	0.45
Albumin (g/dl), median (normal 3.4–5.3)	3.3	3.1	3.5	0.18	3.6	3.5	0.81
Total protein (mg/dl), median (normal 6.0–8.2)	7.1	7.1	7.3	0.79	7.1	7.0	0.92
IgG, Total (mg/dl), median (normal 600–1,640)	2,149	2,319	2,298	0.68	2,137	1769	0.88
IgA (mg/dl), median (normal 47–310)	377	377	343	0.56	504	298	0.18
IgM (mg/dl), median (normal 50–300)	166	136	273	1.00	231	161	0.08
ANA titer (≥1:80), *n* (%)^c^	26 (62)	13 (87)	5 (56)	0.048	2 (40)	6 (50)	0.81
ASMA (≥20 U), *n* (%)^c^	14 (47)	13 (87)	5 (62)	0.21	^d^	1 (9)	^d^

HBV, Hepatitis B virus; HCV, Hepatitis C virus; NASH/NAFLD, Nonalcoholic steatohepatitis/nonalcoholic fatty liver disease; CS, corticosteroids; CNI, calcineurin inhibitor; AZA, azathioprine; MMF, mycophenolate mofetil; mTOR, mammalian target of rapamycin inhibitor; TB, total bilirubin; ANA, Anti-nuclear antibody; ASMA, Anti-smooth muscle antibody.

a Fisher’s exact test.

b All subjects were HBsAg negative. One subject in tnAIH was AMA positive. Two subjects (one PCR, one tnAIH) were HCV Ab positive, HCV RNA in these subjects were negative.

c Percent with data available.

d No data available.

Bolded values represent statistically significant difference (p < 0.05).

Calcineurin inhibitor (CNI) and corticosteroids (CS) were the most common IS at biopsy (47% and 45% of all subjects, respectively) (Table 1). Apart from one subject on a longstanding stable regimen of etanercept for rheumatoid arthritis, all other subjects in the tnAIH had received no IS prior to biopsy. In the fAIH group, most subjects were on a CS-based regimen (70%) ± azathioprine (29%) or CNI (14%). IS use in the rAIH group were varied, with CNI (87%) or mammalian target of rapamycin (mTOR) inhibitor (12%) base with the addition of CS (87%), mycophenolate mofetil (MMF) (37%), and/or azathioprine (12%). Among PCR, the IS at biopsy were most commonly CNI-based (93%) with MMF (71%) and/or CS (50%). All episodes of PCR were managed with addition or increase of CS ± uptitration of CNI.

### Histopathological Comparison of Plasma Cell Hepatitis Subtypes

The H&E stained and trichome stained section of liver needle biopsy specimen were reviewed by a liver pathologist for the aforementioned features (Table 2). In the non-transplant groups, tnAIH demonstrated greater median total lymphocyte count (*p* = 0.01), median total plasma cell count (*p* = 0.003), severity of lobular inflammation (*p* = 0.046), and presence of interface hepatitis (*p* < 0.001) compared to fAIH. In the post-transplant groups, PCR demonstrated higher median number of plasma cells (*p* < 0.001) and proportion of plasma cells relative to total lymphocyte count (*p* < 0.001) compared to rAIH. However, the overall histologic similarities among PCH subtypes did not allow for definitive differentiation based on the histopathological assessment with H&E staining.

**TABLE 2:** Histopathologic characteristics of individuals with plasma cell hepatitis.

Parameter	All subjects	Non-transplant			Post-transplant		
		tnAIH	fAIH	*p*-value	rAIH	PCR	*p*-value
	*N* = 47	*N* = 15	*N* = 10		*N* = 8	*N* = 14	
Lymphocytic Infiltration (cells/tract), median							
Total lymphocytes	137.0	167	105	**0.01**	140	110	0.08
Total plasma cells	37.1	50	20	**0.003**	16	48	**<0.001**
% Plasma cells	30.0	30	20	0.26	10	35	**<0.001**
Fibrosis							
Mild/minimal	24 (51)	6 (40)	3 (30)	1.00^a^	5 (62)	10 (71)	1.00^a^
Moderate/severe	23 (49)	9 (60)	7 (70)		3 (37)	4 (29)	
Portal Inflammation, *n* (%)							
Mild/minimal	9 (19)	1 (7)	1 (10)	1.00^a^	2 (25)	5 (36)	1.00^a^
Moderate/severe	38 (81)	14 (93)	9 (90)		6 (75)	9 (64)	
Lobular inflammation, *n* (%)							
Mild/minimal	30 (64)	4 (27)	7 (70)	**0.046** ^a^	8 (100)	11 (78)	0.27^a^
Moderate/severe	17 (36)	11 (73)	3 (30)		0 (0)	3 (21)	
Perivenular inflammation, *n* (%)							
Mild/minimal	30 (64)	7 (47)	8 (80)	0.14^a^	7 (87)	8 (57)	0.19^a^
Moderate/severe	17 (36)	8 (53)	2 (20)		1 (12)	6 (43)	
Bridging necrosis, present, *n* (%)	11 (23)	8 (53)	2 (20)	0.21^a^	1 (12)	0 (0)	0.12^a^
Interface hepatitis, present, *n* (%)	38 (81)	15 (100)	7 (70)	**<0.001** ^a^	7 (87)	9 (64)	0.58^a^
Perivenular necrosis, present, *n* (%)	17 (36)	8 (53)	2 (20)	0.18^a^	1 (12)	6 (43)	0.19^a^

a Fisher’s exact.

Bolded values represent statistically significant difference (p < 0.05).

### Comparison of IgG4-PC Infiltration

**FIGURE 1:**
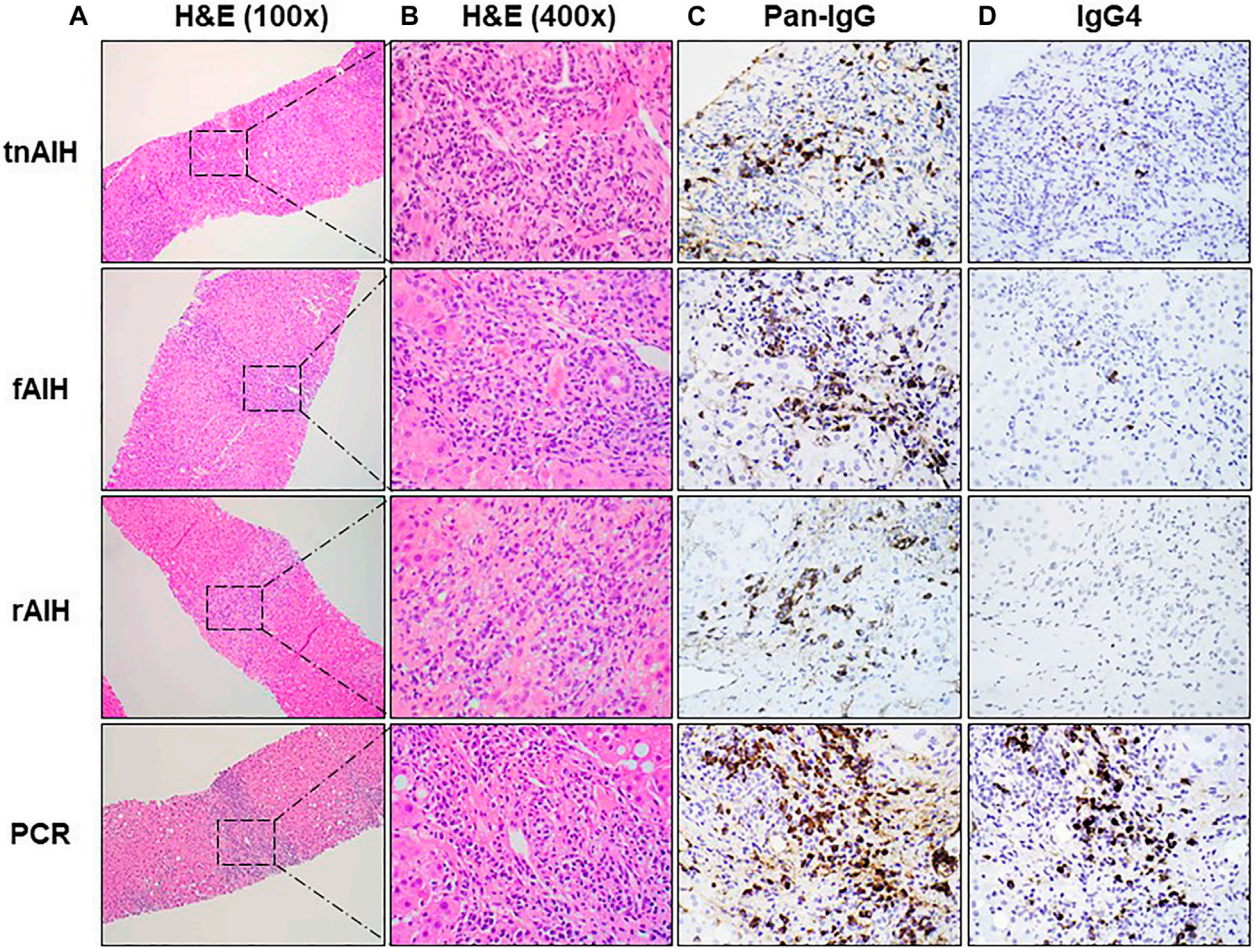
Representative histopathologic and immunohistochemical findings of PCH subtypes: H&E stain of representative portal tract at low magnification (×100) with box with dotted line showing foci of plasma cell infiltration **(A)**, higher magnification (×400) of the selected area of plasma cell aggregates in **(A)** with H&E staining **(B)**, the immunohistochemical (IHC) staining of the representative portal tracts with anti-pan-IgG antibody (×400) **(C)**, and the IHC staining of the corresponding portal tract with anti-IgG4 antibody (×400) **(D)**.

**FIGURE 2:**
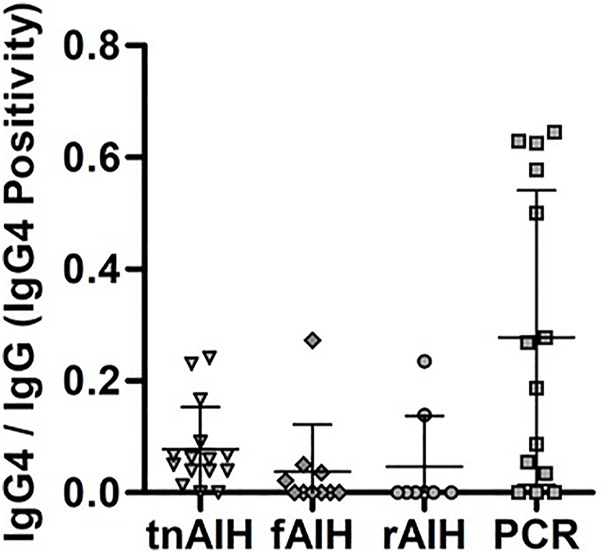
IgG4-Positivity distribution within each PCH subtype. The number IgG4-PC was normalized with the total number of PC in the representative portal tract to determine the IgG4-Positivity. The IgG4-Positivity of each subject are displayed based on their clinical diagnosis: tnAIH (*n* = 15), fAIH (*n* = 10), rAIH (*n* = 8), and PCR (*n* = 14).

To investigate whether each PCH disease reflects distinctive or overlapping immuno-pathologies, we further examined the specimen by immunophenotyping of infiltrating PC via immunohistochemical analysis of IgG4 expression. IgG4-PC was identified in 30 cases (64%), while the remaining 17 cases did not exhibit the presence of IgG4-PC (Table 3). IgG4-PC was not seen in the lobular inflammatory component of any cases. Prevalence of IgG4-PC was highest for tnAIH (87%) followed by PCR (79%), fAIH (40%), and rAIH (25%). The IgG4-Positivity, defined as the number of IgG4-PC over the total number of IgG-expressing PC in the corresponding portal tract, was highly divergent among the PCH types (Figures 1, 2). The diagnosis of PCR demonstrated the highest median IgG4-Positivity (0.228, IQR 0.039–0.558), followed by tnAIH (0.060, IQR 0.040–0.079), rAIH (0.000, IQR 0.000–0.035), and fAIH (0.000, IQR 0.000–0.033).

**TABLE 3:** Prevalence of IgG4-PC in the subtypes of plasma cell hepatitis.

Parameter	All subjects	Non-transplant			Transplant		
		tnAIH	fAIH	*p*-value	rAIH	PCR	*p*-value
	*N* = 47	*N* = 15	*N* = 10		*N* = 8	*N* = 14	
Prevalence of IgG4-PC, *n* (%)							
IgG4-PC Present	30 (64)	13 (87)	4 (40)	**0.03** ^a^	2 (25)	11 (79)	**0.03** ^a^
IgG4-PC Absent	17 (36)	2 (13)	6 (60)		6 (75)	3 (21)	
IgG4-Positivity							
IgG4-PC, median	2	2	0	**0.014**	0	10	**0.01**
IgG-PC, median	35	50	20	**<0.001**	16	43	**<0.001**
IgG4-PC/IgG-PC, median	0.040	0.060	0.000	**0.02**	0.000	0.228	**0.02**
IgG4-PC/IgG-PC, IQR	0.000–0.0177	0.040–0.079	0.000–0.033		0.000–0.035	0.039–0.558	
IgG4-Positivity Tertile							
1	17 (36)	2 (13)	6 (60)	0.05^a^	6 (75)	3 (21)	0.06^a^
2	15 (32)	9 (60)	3 (30)		0 (0)	3 (21)	
3	15 (32)	4 (27)	1 (10)		2 (25)	8 (58)	

IgG4-PC: immunoglobulin G subclass 4-positive plasma cells.

IgG-PC: immunoglobulin G-positive plasma cells.

a Fisher’s exact test.

Bolded values represent statistically significant difference (p < 0.05).

In the non-transplant groups, tnAIH demonstrated a higher median IgG4-Positivity than fAIH (*p* = 0.03). In the transplant groups, PCR demonstrated a higher median IgG4-Positivity than rAIH (*p* = 0.03) (Table 3). Five subjects with IgG4-Positivity ≥0.500 were all in the PCR group (Figure 2). No rAIH subjects demonstrated this degree of IgG4-Positivity. These observations raise the possibility that PCH is comprised of heterogenous immunophenotypes as the IgG4-Positivty is highly variable among and within PCH subtypes.

### Association Between IgG4-Positivity and Clinical Presentation

To better understand the clinical implication of IgG4-PC infiltration in PCH, we evaluated the potential association between IgG4-Positivity, laboratory parameters, histopathology and therapeutic outcomes. Our analysis showed that increasing tertile of IgG4-Positivity was associated with increasing peripheral eosinophil percentage (*p* = 0.02). This is consistent with the findings seen in other IgG4-RD (32). The proportion of subjects with CS use at the time of the biopsy decreased with increasing tertile of IgG4-Positivity (*p* < 0.001). Alternatively, no statistically significant trends were detected between degree of IgG4-Positivity and serum white blood cell count (*p* = 0.71), serum sodium (*p* = 0.50), serum creatinine (*p* = 0.83), ALP (*p* = 0.39), AST (*p* = 0.66), ALT (*p* = 0.64), or total bilirubin (*p* = 0.70) at time of biopsy (Table 4). Notably, no significant association was detected between IgG4-Positivity and serum IgG level (*p* = 0.14) or elevated ANA titer (≥1:40) (*p* = 0.78). ASMA positivity decreased by tertile of IgG4-Positivity from 71% to 36% in the lowest to highest tertiles, although this trend did not achieve statistical significance (*p* = 0.09). The relationship between serum IgG4 level and IgG4-Positivity could not be assessed due to limited data, though prior studies have shown that serum IgG4 concentration does not serve as the surrogate for the degree of IgG4-PC infiltration in the liver (28).

**TABLE 4:** Clinical and histopathologic characteristics stratified by IgG4-positivity.

Parameters		IgG4-positivity			
	Tertile	1	2	3	*p*-value
	IgG4-PC/IgG-PC	0	>0–0.087	>0.087	
	*n*	17	15	15	
Age (years), median		41	50	57	0.11
BMI (kg/m^2^), mean		27.41	31.98	27.62	0.64
Female, *n* (%)		13 (76)	12 (80)	10 (67)	0.54
Family history of autoimmune diseases, *n* (%)		2 (12)	2 (13)	4 (27)	0.27
Immunosuppressants (IS), *n* (%)					
CS		14 (82)	4 (27)	3 (20)	<0.001
CNI		9 (53)	4 (27)	9 (60)	0.74
AZA		2 (12)	0 (0)	1 (7)	0.53
MMF		6 (35)	2 (13)	5 (33)	0.86
MTOR		1 (6)	0 (0)	0 (0)	0.24
Other IS		0 (0)	0 (0)	1 (7)	0.21
Laboratory Data					
Pre-Treatment					
WBC (cells/L x109), median (normal 4.1–10.8)		6.2	6.4	4.9	0.71
Neutrophil %, median (normal 40–60%)		60	54	57	0.48
Lymphocyte %, median (normal 20–40%)		25	30	26	1.00
Eosinophil %, median (normal 0–3%)		1.5	1.8	3.7	0.02
Platelet count (K/cumm), median (normal 141–401)		172	204	148	0.22
Na (mg/dl), median (normal 135–145)		137	137	138	0.50
Cr (mg/dl), median (normal 0.40–1.10)		0.81	0.67	0.87	0.83
ALP (U/L), median (normal 34–106)		199	122	213	0.39
AST (U/L), median (normal 38–126)		98	353	107	0.66
ALT (U/L), median (normal 14–54)		120	297	111	0.64
TB (mg/dl), median (normal 0.2–1)		1.3	4.5	1.2	0.70
Albumin (g/dl), median (normal 3.4–5.3)		3.6	3.8	3.3	0.35
Total protein (mg/dl), median (normal 6.0–8.2)		6.8	7.4	7.0	0.41
IgG, Total (mg/dl), median (normal 600–1,640)		1,617	2,495	2,420	0.14
ANA titer (≥1:80), n (%)^a^		9 (60)	11 (79)	7 (54)	0.78^b^
ASMA (≥20 U), n (%)^a^		10 (71)	8 (67)	4 (36)	0.09^b^
On Treatment					
Day 7–10 (% change from pre-treatment, median)					
ALP		−19.4	−15.5	−15.1	0.92
AST		−40.0	−71.1	−65.9	0.16
ALT		−42.8	−44.9	−60.7	0.19
TB		−13.8	−28.6	−27.8	0.45
Day 30–60 (% change from pre-treatment, median)					
ALP		−23.9	−29.3	−25.0	0.74
AST		−42.4	−78.5	−78.9	0.08
ALT		−43.4	−69.9	−74.6	0.13
TB		−15.9	−68.5	−28.2	0.54
Histopathologic data					
Fibrosis, *n* (%)					
Mild/minimal		7 (41)	7 (47)	10 (67)	0.57^b^
Moderate/severe/cirrhosis		10 (59)	8 (53)	5 (33)	
Portal inflammation, *n* (%)					
Mild/minimal		5 (29)	0 (0)	4 (27)	0.78^b^
Moderate/severe		12 (71)	15 (100)	11 (73)	
Perivenular inflammation, *n* (%)					
Mild/minimal		14 (82)	7 (47)	8 (53)	0.04^b^
Moderate/severe		3 (18)	8 (53)	7 (47)	
Lobular inflammation, *n* (%)					
Mild/minimal		17 (100)	5 (33)	8 (53)	0.005^b^
Moderate/severe		0 (0)	10 (67)	7 (47)	
Interface hepatitis, *n* (%)		8 (47)	13 (87)	11 (73)	0.51^b^
Perivenular necrosis, *n* (%)		3 (18)	8 (53)	3 (20)	0.82^b^
Bridging necrosis, *n* (%)		2 (12)	7 (47)	3 (20)	0.54^b^

IS, immunosuppressants; CS, corticosteroids; CNI, calcineurin inhibitor; AZA, azathioprine; MMF, mycophenolate mofetil; mTOR, mammalian target of rapamycin inhibitor; TB, total bilirubin; ANA, anti-nuclear antibody; ASMA, anti-smooth muscle antibody.

a Percent with data available.

b Fisher’s exact test. Binary comparisons made using “mild/minimal” vs. other where relevant.

Histologically, higher degree of IgG4-Positivity was associated with severe necro-inflammatory change in the histological assessment (Table 4). The proportion of subjects with moderate or severe perivenular inflammation (*p* = 0.04) and lobular information (*p* = 0.005) increased with tertile of IgG4-Positivity but was not significantly associated with presence of moderate or severe fibrosis (*p* = 0.57).

To understand the association between IgG4-Positivity and biochemical response to treatment in the post-LT setting, an analysis comparing responsiveness (percent improvement from index biopsy) of liver-related serologic tests (ALP, AST, ALT, TB) of current diagnoses (rAIH and PCR) to IgG4-Positivity strata (<0.500 and ≥0.500) was performed. High IgG4-Positivity (≥0.500), all of which were PCR cases, was associated with greater improvement of AST (*p* = 0.04) at 7–10 days after treatment initiation. However, no significant difference was found when comparing by diagnosis (*p* = 0.11) (Table 5). The % improvement in AST at later time point (30–60 days) also increased with increasing tertile of IgG4-Positivity, though did not reach statistical significance (*p* = 0.08) (Table 4).

**TABLE 5:** Comparison of treatment response of post-LT PCH stratified by diagnosis and IgG4-Positivity.

Laboratory data	Diagnosis			IgG4-positivity		
	rAIH	PCR	*p*-value	<0.500	≥0.500	*p*-value
	*N* = 8	*N* = 14		*N* = 17	*N* = 5	
Percent change from index biopsy^a^ (median)						
At Day 7–10						
ALP	−11.6	−24.9	0.48	−11.9	−25.4	0.37
AST	−32.7	−65.9	0.11	−36.4	−82.9	**0.04**
ALT	−20.2	−61.0	0.34	−20.0	−62.2	0.11
TB	−0.1	−25.8	0.36	−9.1	−40.0	0.16
At Day 30–60						
ALP	−2.9	−34.5	0.10	−23.9	−27.8	0.57
AST	−31.5	−72.7	0.12	−54.5	−91.1	0.27
ALT	−17.8	−68.8	0.12	−36.4	−79.6	0.41
TB	−7.9	−18.3	0.62	−16.7	+46.7	0.36

a Percent change = (Value at Index Biopsy—Value at Day X)/Value at Index Biopsy.

Bolded values represent statistically significant difference (p < 0.05).

In summary, our analysis suggests that the high degree of IgG4-PC proliferation is associated with the histological features of severe inflammation in non- and post-LT setting, as well as more rapid response to IS in the post-LT setting.

## Discussion

This represents the first study to comprehensively evaluate the degree of IgG4-PC infiltration across types of PCH in the non- and post-transplant setting. Notably, our results showed a high degree of IgG4-PC infiltration more frequently associated with the clinical diagnosis of PCR than tnAIH, fAIH, and rAIH. In particular, markedly high IgG4-Positivity (≥0.500) was only found in PCR cases. This implies that IgG4-PC infiltration to this extent is not a general immune response to a liver allograft, but rather appears to be a unique immunopathological fingerprint of PCR (Figure 2). We also note that there are cases of PCR with minimal or absent of IgG4-PC infiltration. Accordingly, the overlapping profiles of rAIH and some cases of PCR (IgG4-Positivity <0.500) raises the possibility a shared immunopathology in these two separate clinical entities (Figure 2). These findings may support prior hypotheses that a pathophysiologically-distinct *de novo* AIH exists separately from PCR (33). Moreover, there are some cases with relatively high degree of IgG4-PC infiltration (IgG4-positivity >0.20) in AIH cases, especially in tnAIH group. These observations collectively indicate that each PCH disease consists of heterogeneous immunophenotypes. Additionally, we found that differentiation of PCH by IgG4-positivity may be of direct clinical relevance in the post-LT setting. The group with markedly high IgG4-Positivity (≥0.500) appears to demonstrate a more rapid response to directed IS. Consequently, our study suggests that evaluation of IgG4-Positivity may serve as a valuable diagnostic approach in the post-LT setting with corresponding implications in management. Additionally, the lack of rAIH cases with markedly high IgG4-Positivity in our study suggests that IgG4-Positivity may be of diagnostic utility when pre-LT diagnoses is unclear or unknown. Specifically, an IgG4-Positivity threshold of ≥0.500 may effectively rule out a diagnosis of rAIH. This is of potential therapeutic relevance as rAIH has been known to require aggressive immunosuppression to prevent graft loss (14).

To date, there remain substantial gaps in understanding the underlying pathophysiology of PCH, which significantly hinders clinical management of PCH diseases. In particular, there is concern as to whether each PCH disease represents a distinctive condition or a cluster of broader, overlapping conditions than current classification schemes. The latter case might, at least in part, be the basis for the variable degree of response to IS among and within the PCH diseases. Current management strategies of PCH entirely rely on the empiric use of a variety of IS, which may result in significant toxicity and morbidity such as, but not limited to, the development of infection and malignancy. While the current empiric management strategy is capable of inducing remission in a large proportion of patients, the prognosis of individuals with PCH remains highly variable. In the non-LT setting, transplant-free survival of AIH is 91% and 70% at 10 and 20 years from the initial diagnosis, respectively, with up to 40% progressing to cirrhosis despite treatment (18, 34). Similarly, rAIH is responsible for 1% of all deaths at 5 years post-transplantation due to graft failure and is associated with increased risk of death from infection (14). With regards to PCR, the overall prognosis in adults is not well defined, although studies in pediatric populations demonstrate no significant impact on the prognosis (35, 36). Though PCR typically responds well to increased doses of corticosteroids, it is understood that non- or under-treated patients eventually progress to graft loss (13). As use of IS also carries its own risks (19, 37), an establishment of a specific, molecular-targeted therapeutic strategy that abrogates off-target toxicities provides an opportunity to improve patient outcomes.

Given advancement in understanding of IgG4-RD, there has been increasing interest in the involvement of IgG4-PC in liver diseases. The pioneer work examined the liver tissue of autoimmune pancreatitis patients with liver enzyme abnormalities, in which the dense infiltration of IgG4-PC in the liver parenchyma was observed in nearly all cases (28). Since the histopathological findings also exhibited multiple features commonly seen in AIH, further studies investigating the presence of IgG4-PC in the liver tissue of patients with AIH were performed (38–40). These studies found an infiltration of IgG4-PC in a small proportion of cases based on the criteria of >5 or 10 IgG4-PC per high power field (HPF). In contrast, more pronounced degree of IgG4-PC infiltration, >25 IgG4-PC per HPF was noted in cases of PCR (29). While these findings led to substantial excitement into the field, the definition of the “IgG4-PC infiltration” determined in a binary fashion (e.g. “positive” or “negative”) or the number of cells staining positive for IgG4 per HPF might not serve as an objective indicator since it lacks the consideration of the total number of PC aggregated in the corresponding foci. To date, the lack of a standardized evaluation method for IgG4-PC infiltration has severely limited its clinical utility—despite expert acknowledgement of its importance in prior consensus statements (12). To overcome this fundamental issue, we established a rigorous approach to better evaluate the degree of IgG4-PC infiltration. To this end, we determined the frequency of IgG4-PC by normalizing the number of IgG4-PC over the total number of PCs in the corresponding portal tracts using serially sectioned slides (IgG4-Positivity). In addition, we sought to minimize the risk of data interpretation bias resulting from the potential sampling error by enrolling only samples that have at least 8 portal tracts, of which the foci with the representative tracts were used to evaluate the degree of IgG4-PC infiltration. We believe our quantification system allows objective cross-sectional evaluation of IgG4-PC infiltration.

IgG4 is known to have a unique immunomodulatory effect distinct from other types Ig (24). Other than antigen binding, Ig plays an important role in activation of the immune system through two discrete mechanisms: 1)Ig binding to fragment crystallizable (Fc) receptors (FcR) expressed on the cell surface of various immune cells, which augment the cytotoxic and phagocytic function of immune cells, and 2)Ig interaction with complement component 1q (C1q), resulting in activation of classical complement pathway. Of great interest, IgG4 has been considered a noninflammatory IgG compared with other IgG subclasses due to stronger affinity to the inhibitory FcR, Fcγ receptor IIB, and an inferior capacity in complement pathway activation (41, 42). These notions regarding the “non-inflammatory” characteristics of IgG4 appears irrelevant in PCH, at least in our study, as our observations revealed that IgG4-Positivity is associated with higher degree of hepatic necroinflammation. A potential explanation is that IgG4-PCs may serve an anti-inflammatory role when severe inflammation is present. Interestingly, despite the positive association of IgG4-Posivity with inflammation severity, there was no difference with respect to liver biochemistries at biopsy (Table 4).

It has been shown that the production of IgG4 involves an activation of unique upstream immune pathway, in which interleukin (IL)-4 and IL-10 secreted from CD4^+^ (C-X-C chemokine receptor type-5) CXCR5^+^ T cell, namely Tfh cells, facilitates transdifferentiation of naive B cells to IgG4-PC (43–45). Accordingly, affected organs in IgG4-RD exhibit an abundant infiltration of Tfh cells (45, 46). In addition, the clonal expansion of CD4^+^ T cells with a cytotoxic function (CD4^+^ CTLs), which abundantly express SLAM family member 7 (SLAMF7), granzyme A (GZMA), IL-1β, and TGF-β, is observed in the affected organ of IgG4-RD (47, 48). TGF-β is a potent anti-inflammatory cytokine and contributes to the fibrosis development. Thus, development of fibrosis a hallmark feature of IgG4-RD. However, we observed that infiltration of IgG4-PC was not associated with fibrosis severity. This potential discrepancy between IgG4-RD and PCH with IgG4 PC infiltration may be partially due to the close laboratory monitoring of AIH and post-LT patients, resulting in uptitration of IS before the fibrosis development. Alternatively, the immunosuppressive properties of IgG4 and TGF-β might have a stronger impact on disease presentation than the pro-fibrotic effect of TGF-β in PCH.

In general, IgG4-RD is known to be highly responsive to glucocorticoid-based IS. This notion coincides with the immunosuppressive properties of IgG4 as well as the anti-inflammatory effect of TGF-β secreted by the CD4^+^ CTLs. Accordingly, previous studies by others reported that AIH cases with IgG4-PC infiltration were associated with a comparatively rapid response to steroid therapy to the induction of clinical remission (38, 40). Moreover, the relatively high frequency of IgG4-PC seen in PCR cases in our observation as well as by others (29) may serve as an explanation as to why a less aggressive regimen of IS is generally required for PCR compared to AIH and rAIH (8, 49, 50). This notion is also supported by our finding that markedly high IgG4-Positivity in the post-LT setting (≥0.500) was correlated with rapid improvement of serum AST, for which all cases were in the PCR group (Table 5). Moreover, the strong association of decreasing IgG4-Positivity and CS use is also congruent with existing paradigm of steroid-responsiveness in IgG4-RD (Table 4). These observations suggest that evaluating IgG4-Positivity has a direct relevance to clinical management of PCH, particularly in the post-LT setting, as it may be of prognostic value in assessing expected response to corticosteroid therapy.

In summary, this study is the first to cross-sectionally demonstrate the diversity of the immunophenotypic profile of PCH in the non- and post-transplant setting, in the absence and presence of IS, as defined by IgG4-Positivity—with significant differences across and within disease entities. The highly heterogenous IgG4-Positivity across and within PCH entities indicate that current classification of PCH diseases is insufficient in capturing the immuno-pathophysiology. Hence, we propose refining the PCH classification strategy by incorporating the IgG4-Positivity based stratified categorization, particularly in the post-LT setting; which might inform a immunopathology-specific management strategy and prognostication. Toward this goal, further prospective studies with a larger number of subjects treated with a standardized IS regimen, and long-term follow up, are warranted.

## Data Availability

The raw data supporting the conclusion of this article will be made available by the authors, without undue reservation.

## Glossary

AIH: autoimmune hepatitis
ANA: anti-nuclear antibody
ASMA: anti-smooth muscle antibody
AZA: azathioprine
BMI: body mass index
CD4+: cluster of differentiation 4 positive
**CD4^+^** CTLs: CD4^+^ T cells with a cytotoxic function
CNI: calcineurin inhibitor
CS: corticosteroids
CTL: cytotoxic T lymphocyte
CXCR5: C-X-C chemokine receptor type 5
fAIH: flare of autoimmune hepatitis
Fc: fragment crystallizable
FcR: Fc receptor
FFPE: formalin-fixed paraffin embedded
GZMA: granzyme A
H&E: hematoxylin-eosin
Ig: immunoglobulin
IgG4: immunoglobulin G subclass 4
IgG4-PC: IgG4-producing plasma cells
IgG4-RD: IgG4-related disease
IHC: immunohistochemical
IL: interleukin
IS: immunosuppressants
LT: liver transplantation
MMF: mycophenolate mofetil
mTOR: mammalian target of rapamycin
PC: plasma cell
PCH: plasma cell hepatitis
PCR: plasma cell-rich rejection
rAIH: recurrent autoimmune hepatitis
SLAMF7: SLAM family member 7
Tfh: T follicular helper
TGF-β: transcription growth factor-β
tnAIH: treatment-naïve autoimmune hepatitis
